# Study on the intervention effect of HCH integrated SMG health management model on community high-risk group of cardiovascular disease

**DOI:** 10.3389/fcvm.2024.1399787

**Published:** 2024-07-15

**Authors:** Rui Du, Kaifang Ma, Yanru Li, Jin Tian, Mengkun Li, Chenxi Zhao, Jing Wang

**Affiliations:** ^1^ School of Public Health, Hebei Medical University, Shijiazhuang, China; ^2^ Department of Public Health, The First Hospital of Hebei Medical University, Shijiazhuang, China; ^3^ Hebei Key Laboratory of Nutrition and Health, School of Public Health, Hebei Medical University, Shijiazhuang, China

**Keywords:** high-risk populations with cardiovascular disease, Hospital-Community-Home, Self-Mutual-Group health management model, self-efficacy, cardiovascular disease

## Abstract

**Objective:**

This study aimed to evaluate the integration of the Hospital-Community-Home (HCH) model with the Self-Mutual-Group (SMG) health management model for high-risk populations with cardiovascular disease in the Yuhua community of Shijiazhuang city. The study focused on implementing care interventions (HCH, SMG) with a specific emphasis on SMG to promote beneficial views/behaviors, enhance self-efficacy/agency, and address detrimental determinants of health, ultimately leading to durable changes and healthier lifestyles. Comparing the HCH model with the combined SMG model helps to comprehensively assess the strengths and weaknesses of different health management approaches. This comparison contributes to theoretical innovation and practical development in the field of health management, as well as improving patients' health outcomes and quality of life.

**Methods:**

This study employed a quasi-experimental design. Using stratified sampling, individuals who underwent health examinations in Community A and Community B from Shijiazhuang city between May 2023 and August 2023 were randomly selected. After informing the participants about the study and obtaining informed consent via telephone, high-risk patients with cardiovascular disease were screened based on their medical examination reports. Data on lifestyle behaviors, self-efficacy, medical responses, quality of life, and readmission rates were collected and compared before and after the intervention.

**Results:**

A total of 526 eligible participants were included, with 241 in the control group and 285 in the study group. After the intervention, there was no significant change in the proportions of smokers, alcohol consumers, and individuals engaging in leisure exercises in the control group. However, in the study group, the proportions of smokers and alcohol consumers significantly decreased, while the proportion of individuals engaging in leisure exercises significantly increased. After the intervention, both the study group and the control group showed significant increases in scores on the General Self-Efficacy Scale—Schwarzer (GSES) and the Seattle Angina Questionnaire (SAQ), with the study group scoring significantly higher than the control group. Avoidance and surrender scores significantly increased after the intervention, with the study group scoring significantly lower than the control group. Confrontation scores significantly increased after the intervention, with the study group scoring significantly higher than the control group. During the follow-up period, the study group had a significantly lower readmission rate than the control group.

**Conclusion:**

The integration of HCH with SMG health management model can significantly improve lifestyle behaviors, optimize medical responses, enhance self-efficacy and quality of life, and significantly reduce readmission rates among high-risk populations with cardiovascular disease.

## 1 Introduction

Cardiovascular diseases (CVDs) encompass a spectrum of heart and vascular disorders, including coronary heart disease, peripheral arterial disease, rheumatic heart disease, congenital heart disease, deep vein thrombosis, and pulmonary embolism. With a staggering global mortality rate, CVDs consistently surpass all other causes of death (1). However, many CVDs can be prevented through population-wide strategies that target modifiable risk factors such as tobacco use, unhealthy diet and obesity, physical inactivity, and harmful alcohol consumption (2). This study is more applicable to the Chinese CVD population, as it is based on the characteristics of the Chinese CVD population, which also provide strong support for the applicability of this model (3). CVDs are chronic conditions that typically develop slowly, often remaining asymptomatic for extended periods before symptoms manifest in advanced stages. Primary healthcare settings play a crucial role in assessing and monitoring CVD risk factors, such as elevated blood pressure, blood glucose levels, blood lipids, as well as overweight and obesity. Moreover, there is a significant aging trend and regional development imbalance (4, 5). These characteristics make it challenging for traditional HCH models to meet the needs of all population groups. However, the SMG model can help overcome this limitation by encouraging patient self-management and collaborative support, thereby improving the efficiency and coverage of health management.

China's healthcare service system and healthcare system are continuously improving and developing. The government and society have increasingly emphasized the importance of health management, providing strong support for the implementation of the HCH combined with the SMG model. Additionally, with the rapid development of information technology, emerging formats such as telemedicine and internet healthcare have also provided technological support and convenient conditions for the promotion of this model.

The Hospital-Community-Home (HCH) model, serving as a continuum of care, aims to facilitate a seamless transition for patients from hospital settings to community or home environments while ensuring specialized care (6). In recent years, this care model has gained significant recognition for its ability to meet the ongoing healthcare needs of patients. By addressing the limitations of inadequate family caregiver capacity and simplified periodic hospital follow-ups, the HCH model fosters effective integration among hospitals, communities, and homes. It provides standardized and specialized rehabilitation guidance to patients, promoting disease recovery and enhancing their overall quality of life (7, 8). Shared Medical Governance (SMG) represents an innovative management approach that combines self-management, team-based care, and mutual assistance to empower patients in managing their own health and enhancing their sense of self-efficacy (9). The SMG model emphasizes the patients' proactivity and autonomy in health management (10). In this model, patients not only actively participate in their own health management but also share experiences and provide mutual support through mutual aid and group activities, leading to more effective improvements in health conditions. This model helps break the passive treatment paradigm in traditional healthcare systems, enhancing patients' health literacy and self-management abilities (11). This study aims to compare the HCH model with the combined SMG model to comprehensively assess the strengths and weaknesses of different health management approaches. While the HCH model typically focuses on the continuity of care from healthcare institutions to homes and communities, the inclusion of SMG elements allows for further examination of the long-term impact of this model on patients' self-management abilities and health conditions (12). By conducting comparative research, differences among the models in patient engagement, health improvement outcomes, and healthcare resource utilization efficiency can be revealed, providing scientific evidence for policy-making and practical applications.

Furthermore, with the development of society and the increasing aging population, chronic diseases and other health issues have become more prominent, demanding innovation and research in health management models. As an emerging concept in health management, the SMG model holds great potential for development. Through in-depth research and exploration, this model can be further refined and optimized to better meet the health needs of modern society.

This study explores the implementation of Shijiazhuang city Yuhua community intervention model that integrates SMG health management within the HCH framework for individuals at high risk of CVDs. The SMG model is a potential key intervention measure by synergizing the strengths of both models, this integrated approach aims to provide comprehensive and patient-centered care, effectively addressing the unique needs of individuals at high risk of CVDs. The ultimate goal is to improve health outcomes and enhance overall well-being through a holistic and proactive approach to cardiovascular disease management.

## 2 Materials and methods

### 2.1 Participants

This study employed a quasi-experimental design. A stratified sampling method was utilized to randomly select participants from Community A and Community B in Shijiazhuang city who underwent medical examinations between May 2023 and August 2023. According to the principle of multiple linear regression, the sample size (*n*) should be at least 5 to 10 times the number of independent variables (m) in the equation. The sample size calculation formula is m × (5 to 10) × (1 + 20%). Considering the sensitive nature of the survey content involving patient privacy, non-compliance of patients may occur. Therefore, an additional 5% is added to the original calculation as a contingency, resulting in a final sample size of 200–310 cases. Following initial contact through telephone communication, participants were informed about the study objectives and provided with detailed information to obtain their informed consent. Individuals identified as being at high risk for CVDs based on their medical examination reports were included as study subjects.

### 2.2 Inclusion and exclusion criteria

#### 2.2.1 Inclusion criteria

• Age range of 35–75 years, with no gender restrictions.
• Compliance with the high-risk criteria for cardiovascular diseases outlined by the World Health Organization (WHO) guidelines, including a 10-year CVDs risk exceeding 20%, hypertension, dyslipidemia, and other relevant factors.
• Adequate cognitive and linguistic abilities to understand and complete questionnaires and engage in follow-up assessments.
• Permanent residents of the respective project sites, with a minimum residency duration of 12 months subsequent to enrollment.
• Patients are provided with medication guidance and advised to take their medications regularly.
• Voluntary participation in the study after being fully informed about the research purpose and procedures.

#### 2.2.2 Exclusion criteria

• Coexistence of psychiatric or neurological disorders that may impair communication and comprehension abilities.
• Presence of severe cardiovascular diseases.
• Concurrent diagnosis of autoimmune diseases, malignant tumors, or other debilitating conditions that significantly affect quality of life.
• Severe liver or kidney dysfunction.

### 2.3 Intervention measures

HCH Intervention: The medical institution group was responsible for the diagnosis and treatment of CVDs and provided guidance on family care and community care for CVDs prevention. The hospital carries out health publicity and education on disease hazards, prevention and health care measures, etc. online (official account, Tiktok, etc.) and offline (health lecture hall, talk health, etc.); Pay attention to medication, diet, lifestyle, and psychological aspects every two months, and adjust the next intervention plan. Family care primarily involved providing daily life assistance, functional exercises, and medication management under medical supervision for individuals with cardiovascular diseases. Community care services, in combination with family care, offered comprehensive and effective long-term care for the older adult. These services included establishing health records, rehabilitation therapy training, medication guidance, professional care, disease screening, regular assessments, and providing psychological support. Follow up on lifestyle habits, diet, behavior, and psychological aspects every month, and provide feedback on the effectiveness of hospital interventions. Family: Self supervision or family supervision of diet, lifestyle behavior, and psychological aspects.

SMG Intervention: SMG intervention was implemented in three levels, characterized by its holistic and progressive nature, allowing patients to gradually accept and adapt to the intervention. The specific implementations were as follows:

(1) Self-management level: The intervention duration was 12 months, and an intervention method can be scheduled to be executed every two weeks or every week, conducted on an individual basis. At the beginning of the intervention, patients were guided to assess their existing health problems, identify pressing issues, and collaboratively establish rehabilitation goals. Self-management aimed to cultivate patients' awareness and abilities in self-health management, including self-care consciousness, proactive medical awareness, self-health assessment skills, and the use of self-help medical devices. The responsibility for health was transferred to the patients themselves, fostering a positive attitude and confidence in understanding and addressing their health problems, thereby enhancing patients' disease management abilities.
(2) Mutual assistance level: The intervention duration was 12 months, and an intervention method can be scheduled to be executed every two weeks or every week, conducted in pairs. Family members were encouraged to actively participate in daily rehabilitation training. Mutual assistance groups were formed based on factors such as age, gender, common interests, rehabilitation goals, and residential communities. Contact was established through phone calls, WeChat, and other means. During each rehabilitation exercise, members of the mutual assistance group could arrange to participate together. If any personal examinations or treatments prevented them from attending rehabilitation as planned, at least four mutual assistance activities were guaranteed each week. The mutual assistance management emphasized strengthening the cohesion between group members, providing necessary support for each other's daily life and emotional well-being, and fostering mutual supervision, encouragement, and support during the rehabilitation process. Peer support was utilized to stimulate patients' ongoing engagement in rehabilitation training and cultivate their awareness of mutual assistance in health management.
(3) Group management level: The intervention duration was 12 months, and an intervention method can be scheduled to be executed every two weeks or every week, conducted with three or more participants as a group. Groups were formed based on disease severity, place of residence, and individual characteristics. Building on the interventions implemented in the previous two levels, group health management was carried out. Group management facilitated patients' integration into social groups beyond their families, allowing them to establish rehabilitation confidence and enhance their sense of self-social value. During the activities, researchers paid special attention to the participation of each patient, mobilizing their enthusiasm for involvement, ensuring the benefits for every group member, and ensuring the steady progress of group management. This level aimed to cultivate awareness and abilities in group health management.

Community A served as the control group, receiving HCH management alone, while Community B served as the study group, receiving integrated HCH and SMG management. The intervention duration for both groups was 6 months.

### 2.4 Evaluation indicators

The investigators, health management team, and community doctors have all undergone unified training, and quality controllers review each link.

(1) Lifestyle behaviors: This includes smoking status (whether the individual currently uses any tobacco products daily or occasionally), alcohol consumption (individuals who currently consume alcohol and have a drinking amount of ≥1 unit per occasion are considered drinkers, with 1 unit being equal to 17 ml of pure alcohol), and participation in physical exercise (individuals who engage in physical exercise at least once a month in the past year are considered participants in physical exercise).
(2) Self-efficacy: The General Self-Efficacy Scale (GSES), developed by Schwarzer, is used to assess self-efficacy before and after the intervention. The GSES consists of 10 items, scored on a 4-point scale. For each item, participants select a response that reflects their actual situation, with 1 indicating “not at all correct,” 2 indicating “a little correct,” 3 indicating “mostly correct,” and 4 indicating “completely correct.” The total score on the GSES ranges from 10 to 40, with higher scores indicating better self-efficacy.
(3) Medical coping styles: The Medical Coping Modes Questionnaire (MCMQ) is used to assess patients' coping styles in response to their illness and medical condition. The MCMQ consists of 20 items organized into three dimensions: Confrontation, Compliance, and Avoidance. Each item is scored on a 4-point scale, with a total score ranging from 20 to 80. Among the 20 items, 12 are positively scored, while items 1, 4, 9, 10, 12, 13, 18, and 19 are reverse scored. A higher score on a particular dimension indicates a greater tendency to adopt that medical coping style.
(4) Current quality of life: The Seattle Angina Questionnaire (SAQ) is used to assess patients' quality of life. The questionnaire covers five dimensions: angina stability, physical limitation, angina frequency, disease perception, and satisfaction with treatment. The total score on the SAQ is 100, with higher scores indicating better quality of life.
(5) Hospitalization rate: The number of hospitalizations due to cardiovascular events during the follow-up period is recorded.

### 2.5 Statistical analysis

Continuous data in this study are presented as mean ± standard deviation (x ± *s*. Statistical analysis for continuous variables is performed using *t*-tests. Categorical data are presented as frequencies (*n*, %), and statistical analysis for categorical variables is performed using chi-square tests. SPSS 22.0 is used for statistical analysis, and GraphPad Prism is used for data visualization. Two-tailed tests are used, and a significance level of *α* = 0.05 is applied to determine statistical significance.

## 3 Results

### 3.1 Baseline characteristics comparison

A total of 526 eligible subjects were included in the study, with 241 in the control group and 285 in the study group. In the control group, there were 132 males and 109 females, with an average age of (56.96 ± 12.63) years and a body mass index (BMI) of (25.36 ± 5.19) kg/m^2^. There were 132 cases of hypertension, 85 cases of diabetes, and 106 cases of hyperlipidemia in the control group. Among them, 89 had a bachelor's degree or higher education, while 152 had a college degree or lower. There were 108 smokers, 93 drinkers, and 120 participants in physical exercise in the control group. In the study group, there were 162 males and 123 females, with an average age of (55.17 ± 15.91) years and a BMI of (26.17 ± 4.83) kg/m^2^. There were 138 cases of hypertension, 106 cases of diabetes, and 131 cases of hyperlipidemia in the study group. Among them, 95 had a bachelor's degree or higher education, while 190 had a college degree or lower. There were 125 smokers, 125 drinkers, and 126 participants in physical exercise in the study group. The general characteristics of the two groups of subjects showed no statistically significant differences (all *P* > 0.05) (Table 1).

**Table 1:** Baseline characteristics comparison.

	Control group	Study group	t/*χ*2	*P*
N	241	285		
Gender			0.227^a^	0.477
Male	132	162		
Female	109	123		
Age ($\bar{x}$ ± *s*, years)	56.96 ± 12.63	55.17 ± 15.91	1.411^b^	0.159
Body mass index ($\bar{x}$±*s*, kg/m^2^)	25.36 ± 5.19	26.17 ± 4.83	1.852^b^	0.065
Hypertension			2.108^a^	0.147
Yes	132	138		
No	109	147		
Diabetes			0.209^a^	0.648
Yes	85	106		
No	156	179		
Hyperlipidemia			0.207^a^	0.649
Yes	106	131		
No	135	154		
Education level			0.743^a^	0.389
Bachelor's degree and above	89	95		
College degree and below	152	190		
Smoking (n)			0.390^a^	0.532
Yes	108	120		
No	133	165		
Alcohol Consumption (Yes/No)			1.495^a^	0.222
Yes	93	125		
No	148	160		
Physical Exercise (Yes/No)			1.634^a^	0.201
Yes	120	126		
No	121	159		

^a^ represents the chi-square value.

^b^ represents the *t*-test value.

### 3.2 Comparison of lifestyle behaviors

Before the intervention, in the control group, there were 108 smokers, 93 drinkers, and 120 participants in physical exercise. In the study group, there were 120 smokers, 125 drinkers, and 126 participants in physical exercise. After 6 months of intervention, lifestyle changes were assessed through questionnaire surveys. In the control group, there were 90 smokers, 80 drinkers, and 137 participants in physical exercise. In the study group, there were 93 smokers, 91 drinkers, and 163 participants in physical exercise. The proportions of smokers, drinkers, and participants in physical exercise showed no significant changes in the control group (all *P* > 0.05). In the study group, the proportions of smokers and drinkers significantly decreased, while the proportion of participants in physical exercise significantly increased (all *P* < 0.05) (Table 2).

**Table 2:** Comparison of lifestyle behaviors.

	Before intervention	After intervention	*χ*2	*P*
Control group				
Smoking	44.8%(108/241)	37.3%(90/241)	2.188	0.139
Alcohol consumption	38.6%(93/241)	33.2%(80/241)	1.524	0.217
Physical exercise	49.8%(120/241)	56.8%(137/241)	2.409	0.121
Study group				
Smoking	42.1%(120/285)	32.6%(93/285)	5.465	0.019
Alcohol consumption	43.9%(125/285)	31.9%(91/285)	8.617	0.003
Physical exercise	44.2%(126/285)	57.2%(163/285)	9.609	0.002

### 3.3 Comparison of self-efficacy

**Figure 1:**
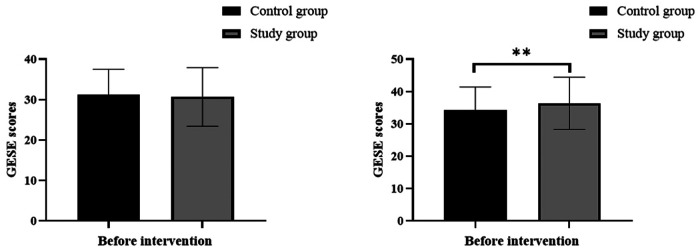
Comparison of self-efficacy, **indicates *P* < 0.01.

Before the intervention, the GSES scores in the control group and study group were 31.21 ± 6.26 and 30.69 ± 7.26, respectively. After the intervention, the GSES scores in the control group and study group were 34.21 ± 7.17 and 36.36 ± 8.07, respectively. Before the intervention, there was no significant difference in GSES scores between the two groups (all *P* > 0.05). After the intervention, the GSES scores significantly increased in both groups, and the study group had significantly higher scores than the control group (all *P* < 0.05) (Figure 1).

### 3.4 Comparison of medical coping styles

Before the intervention, there were no significant differences in the dimensions of confrontation, compliance, and avoidance in medical coping styles between the two groups of subjects. After the intervention, the scores for avoidance and compliance significantly increased, and the study group had significantly lower scores than the control group. The score for confrontation significantly increased, and the study group had significantly higher scores than the control group (all *P* < 0.05) (Table 3).

**Table 3:** Comparison of medical coping styles.

	N	Confrontation (score)		Avoidance (score)		Compliance (score)	
		Before intervention	After intervention	Before intervention	After intervention	Before intervention	After intervention
Control group	241	10.82 ± 2.92	12.39 ± 3.58	17.95 ± 3.28	15.26 ± 3.62	12.95 ± 2.39	11.63 ± 2.67
Study group	258	10.27 ± 3.36	14.92 ± 3.22	18.05 ± 3.11	13.17 ± 3.12	13.08 ± 3.21	9.19 ± 2.41
*t*		1.946	8.310	0.350	6.931	0.510	10.73
*P*		0.052	<0.001	0.727	<0.001	0.610	<0.001

### 3.5 Comparison of quality of life

**Figure 2:**
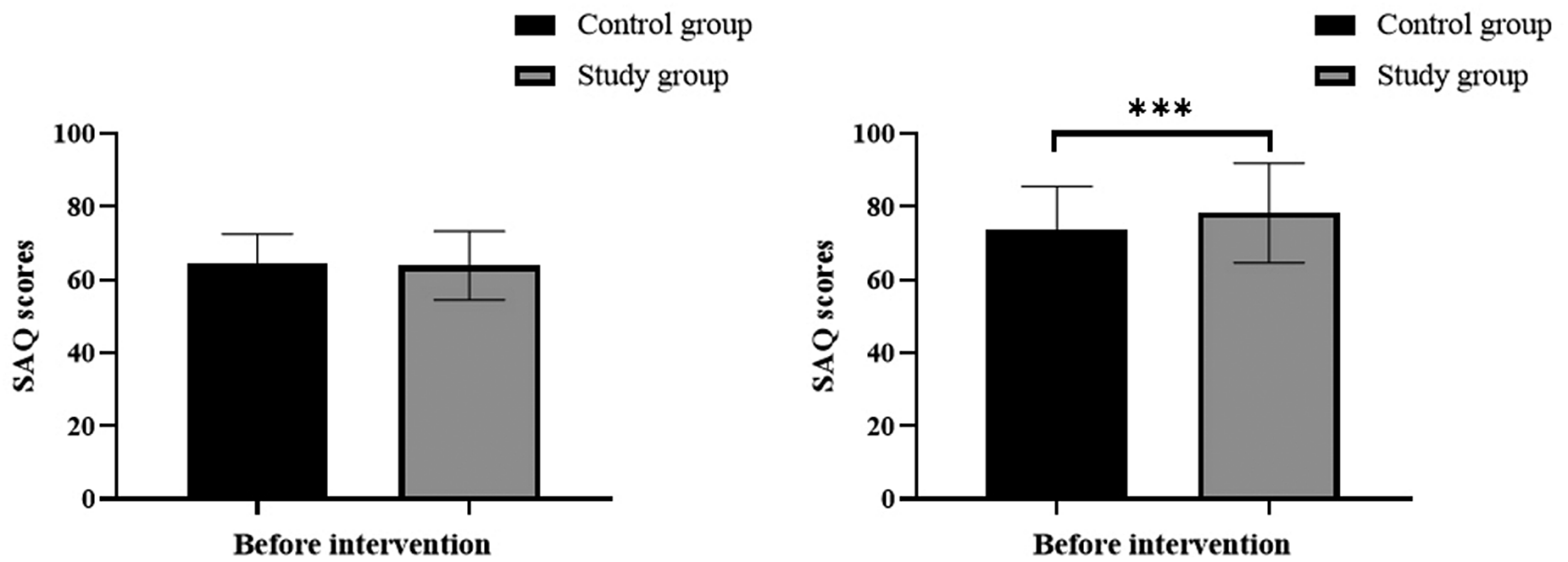
Comparison of quality of life, *** indicates *P* < 0.001.

The SAQ was used to evaluate the quality of life of the subjects before and after the intervention. Before the intervention, the SAQ scores in the control group and study group were 64.32 ± 8.15 and 63.85 ± 9.39, respectively. After the intervention, the SAQ scores in the control group and study group were 73.52 ± 11.96 and 78.28 ± 13.61, respectively. Before the intervention, there was no significant difference in SAQ scores between the two groups (all *P* > 0.05). After the intervention, the SAQ scores significantly increased in both groups, and the study group had significantly higher scores than the control group (all *P* < 0.05). The results are shown in Figure 2.

### 3.6 Comparison of hospitalization rate for cardiovascular events

**Figure 3:**
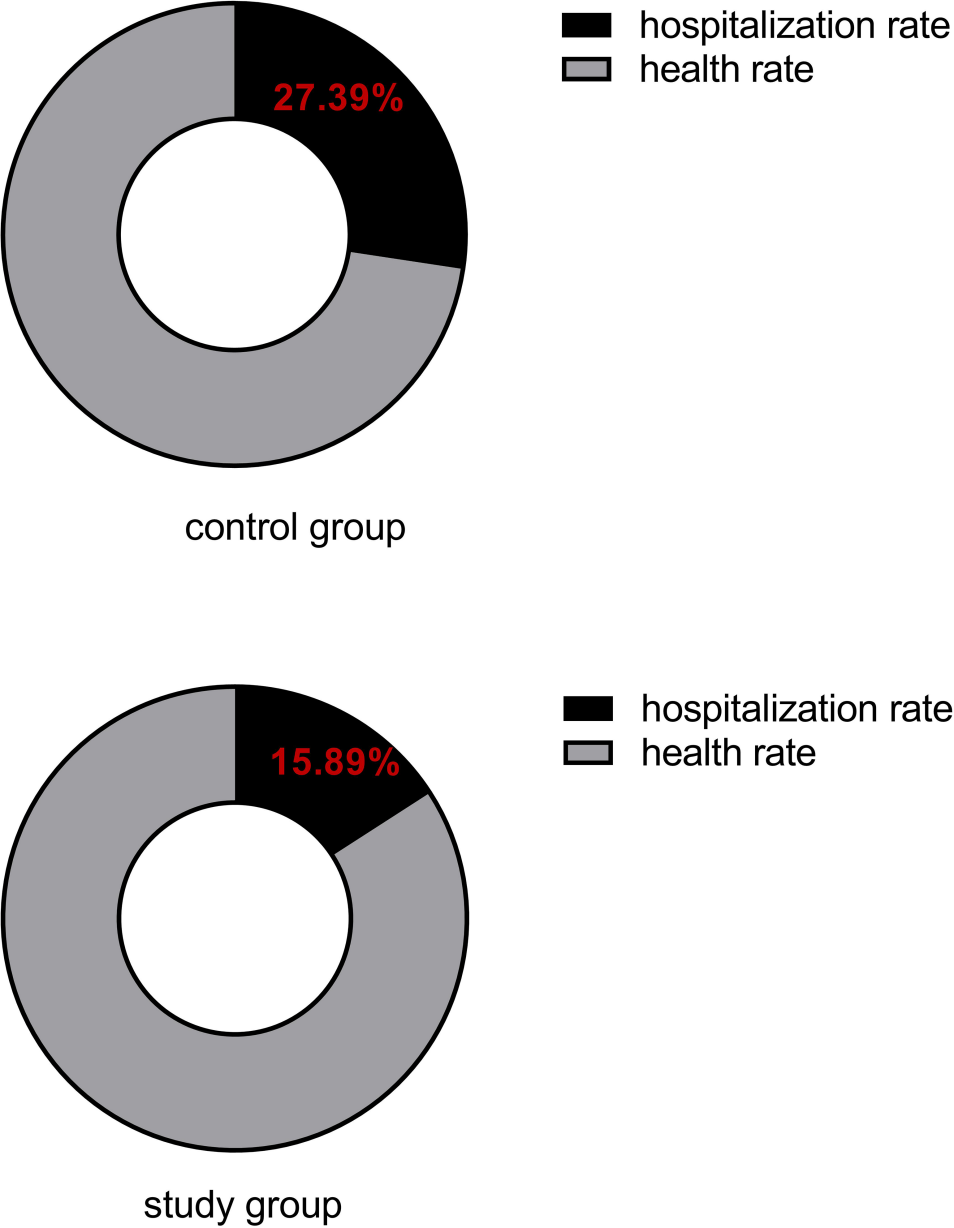
Comparison of hospitalization rate for cardiovascular events.

The number of hospitalizations due to cardiovascular events within 12 months after the intervention was collected for both groups of subjects, as shown in Figure 3. In the control group, there were 66 hospitalizations, with a hospitalization rate of 27.39% (66/241). In the study group, there were 41 hospitalizations, with a hospitalization rate of 15.89% (41/258). The hospitalization rate in the study group was significantly lower than that in the control group (*P* < 0.05).

## 4 Discussion

CVDs stand as a prominent global cause of mortality and disability, necessitating a comprehensive understanding of its risk factors. Extensive epidemiological research has identified a multitude of risk factors, which can be classified into modifiable and non-modifiable categories. Non-modifiable risk factors, including age, sex, genetics, and race, are beyond individuals' control (4, 11, 12). Of particular significance, age serves as an unmodifiable risk factor for CVDs, as the likelihood of developing CVDs escalate with advancing age (13). Conversely, modifiable risk factors encompass smoking, history of high blood pressure or diabetes, obesity, unhealthy diet, physical inactivity, excessive alcohol consumption, elevated blood lipids, and psychosocial factors. These factors, such as excessive alcohol consumption, smoking, high blood pressure, high body mass index, high cholesterol, high blood sugar, poor dietary choices, and sedentary lifestyle, contribute to a substantial portion of global CVDs deaths (14, 15). The present study aimed to compare the intervention effects of SMGs with the HCH model among high-risk populations with CVDs in the community. The findings demonstrated that the integrated SMGs yielded significant improvements in lifestyle, optimized medical responses, enhanced self-efficacy, improved quality of life, and substantially reduced hospitalization rates, surpassing the outcomes achieved by the HCH model.

Self-efficacy, rooted in Bandura's social cognitive theory, encompasses two dimensions: everyday activity efficacy and self-management efficacy. This multifaceted construct reflects individuals' beliefs in their capacity to adapt to their environment and engage in specific behaviors, subtly influencing their behavioral choices and attitudes. Heightened levels of self-efficacy correlate with a stronger conviction in successfully executing behaviors and attaining established goals (16, 17). A robust sense of self-efficacy facilitates the adoption of evidence-based behaviors that mitigate the risks of cardiovascular disease, such as smoking cessation, reduced salt intake, increased consumption of fruits and vegetables, regular physical exercise, and avoidance of harmful alcohol use (18). Medical responses encompass the psychological, cognitive, and behavior-oriented strategies and methods exhibited by individuals in medical settings when confronted with medical stressors. Positive medical responses empower patients to confront their diseases courageously and enhance their quality of life through active rehabilitation treatments (19). Diabetes, high blood pressure, high blood lipids, and other factors independently contribute to cardiovascular disease risk, with pharmaceutical interventions playing a crucial role in reducing cardiovascular risk and preventing cardiac events (20, 21). Reasonable medical responses facilitate timely medical care-seeking, improve medication adherence, delay disease progression, and mitigate the risk of adverse events.

Presently, several health management models cater to high-risk populations with CVDs. These models encompass self-management, community-based approaches, family-based interventions, multidisciplinary strategies, integrated traditional Chinese and Western medicine, HCH tripartite linkage, and network-based health management models. Among these, the HCH model has played a significant role (22, 23). However, with the exception of the self-health management mode, patients often assume a passive role in other models, with decisions regarding disease rehabilitation primarily made by families and medical institutions. Health management models have not fully harnessed the potential for mutual assistance among high-risk populations with cardiovascular disease, and there is a lack of effective integration and application of different models. The SMG model emerges as a comprehensive and multi-level health management approach that emphasizes strong integration. The SMG model delegates all aspects of health management to the patients themselves and utilizes interpersonal communication activities to motivate and enhance their confidence and determination for rehabilitation. This approach facilitates the establishment and maintenance of long-term recovery lifestyles (24, 25). Notably, Lee et al. discovered that team transformation, patient education, self-management, and improved patient communication can effectively enhance healthcare quality, significantly improve patients' sense of health responsibility, and cultivate healthy behaviors (26). The SMG model has found successful application in various settings, including the older adult population living alone, health examination populations, coronary heart disease patients undergoing percutaneous coronary intervention (PCI), and individuals with mental illness. It has demonstrated improvements in self-efficacy and has progressively been integrated into chronic disease management. Its effectiveness in community management of high-risk populations with c CVDs has also been substantiated (27–29).

Although SMG may be resource-intensive, it still remains highly practical and feasible through proper planning, management, and utilization of resources, as well as customization and optimization based on specific contexts (27). SMG requires relatively substantial resource investments, including but not limited to educational materials, training costs, coordinating personnel, and technological support. Additionally, the implementation of this model heavily relies on patient engagement and motivation, which may necessitate additional incentives and measures. However, this does not imply a low feasibility of the model. On the contrary, through proper planning and budget allocation, factors such as patient characteristics and needs (such as age, disease type, health literacy) need to be considered as they influence the acceptance and effectiveness of the model. Furthermore, the support and collaboration of the healthcare system are crucial for the successful implementation of the model. Effective integration and alignment with the existing healthcare service system should also be considered to ensure coherence and synergy (28).

The overall cost of implementing this model is directly related to the clinical environment. Different clinical environments, such as hospitals, communities, and homes, have varying resource conditions, staffing arrangements, and patient needs, which affect the implementation costs of the model. Therefore, effective resource management and utilization based on specific contexts are necessary to enhance its practicality and feasibility when implementing SMG.

## 5 Conclusion

In conclusion, the integration of the HCH model with SMGs represents a comprehensive and patient-centered approach that yields remarkable benefits. This integrated approach results in substantial improvements in lifestyle, optimized medical responses, enhanced self-efficacy, improved quality of life, and a notable reduction in hospitalization rates. By empowering individuals to take charge of their health and promoting effective self-care practices, this model fosters a sense of confidence and control over their condition. As a result, individuals experience a higher quality of life characterized by improved physical well-being, emotional resilience, and overall satisfaction. The integration of the HCH model with SMGs represents a significant advancement in the field of cardiovascular disease management, offering a comprehensive strategy that addresses the diverse needs of high-risk populations in a patient-centered manner.

## Data Availability

The original contributions presented in the study are included in the article/Supplementary Material, further inquiries can be directed to the corresponding author.
